# There are no three physiological narrowings in the upper urinary tract: a new concept of the retroperitoneal anatomy around the ureter

**DOI:** 10.1007/s11604-020-01080-7

**Published:** 2021-01-09

**Authors:** Minobu Kamo, Taiki Nozaki, Saya Horiuchi, Natsuka Muraishi, Jin Yamamura, Keiichi Akita

**Affiliations:** 1 Department of Diagnostic and Interventional Radiology and Nuclear Medicine, University Medical Center Hamburg-Eppendorf (UKE), Martinistrasse 52, 20246 Hamburg, Germany; 2 Department of Radiology, St. Luke’s International Hospital, Chuo-ku Akashi-cho, Tokyo, 104-8560 Japan; 3 Department of Clinical Anatomy, Tokyo Medical and Dental University, 1-5-45 Yushima, Bunkyo-ku, Tokyo, 113-8510 Japan

**Keywords:** Ureter, Urinary stone, Anatomy, Computed tomography, Crossing point, Retroperitoneal space

## Abstract

The widely held dogma of three physiological narrowings in the upper urinary tract has proven incorrect by recent several studies using computed tomography images. There are only two common obstruction sites: the upper ureter and the ureterovesical junction. The second narrowing, where the ureter crosses the iliac vessels, cannot be regarded anymore as a common obstruction site. The mechanism by which stones lodge in the upper ureter is explained anatomically by the change in ureteral mobility and compliance at the level where the ureter exits the perirenal space. This level can be identified radiologically as the point where the ureter crosses under the ipsilateral gonadal veins, termed the “crossing point”. Kinking of the upper ureter is another manifestation of this anatomical phenomenon, visible in radiological images. It is caused by loosening of the ureter at or above the crossing point (within the perirenal space), corresponding with renal descent such as during the inspiratory phase. This new anatomical discovery in the retroperitoneum will not only bring about a paradigm shift in terms of the physiological narrowings in the upper urinary tract, but may also lead to the development of new surgical concepts and approaches in the area.

## Introduction

Most doctors have learned in their medical-school days that there are three physiological narrowings in the upper urinary tract. However, with the development of medical imaging technologies and its application in the field of clinical anatomy, this widely held dogma has recently proven false.

In this review, we first summarize the conventional anatomical concept of the upper urinary tract. Thereafter, we present the renewed concept of upper urinary-tract narrowings, in light of recently discovered anatomical facts concerning the retroperitoneum. Finally, we explain the significance of this novel concept from radiological and clinical aspects.

## Conventional concept

**Fig. 1:**
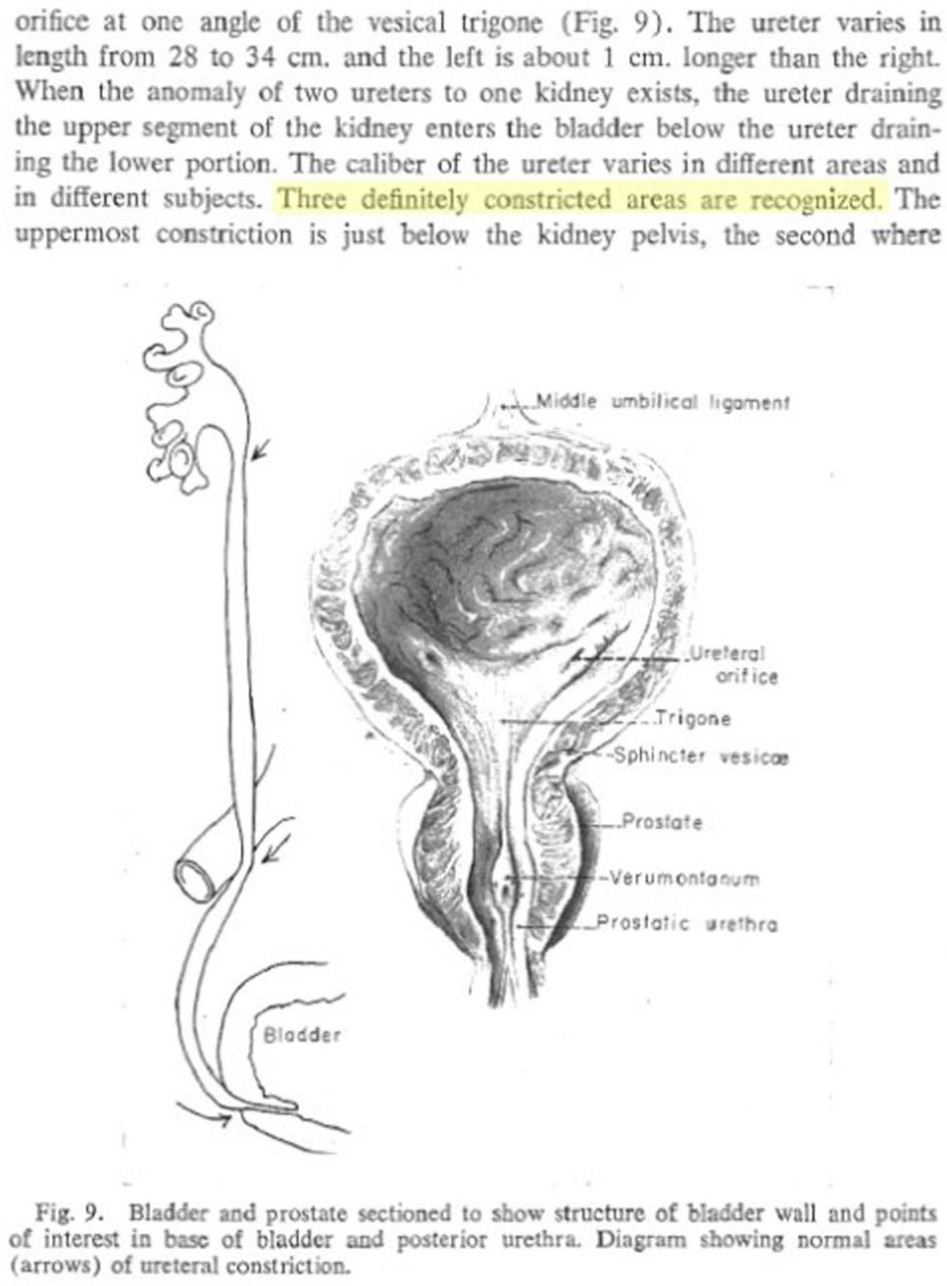
The first publicized description of three physiological narrowings in the upper urinary tract, in 1954 (Dodson AI. Anatomy and surgical approach to the urogenital tract in the male. In: Campbell M, ed. *Urology*. Philadelphia and London: WB Saunders Company; 1954:11–17) [6]. No reference was provided for this description

“The ureter is naturally narrowed at the ureteropelvic junction, at the iliac vessel crossover, and at the ureterovesical junction” [1], “The ureters are constricted at the ureteropelvic junction, pelvic inlet, and bladder entrance” [2]. These are not the only textbooks to describe physiological narrowings in the upper urinary tract; such descriptions can be found in every textbook of anatomy, surgery, or urology [3–5]. This description dates back to the textbook, “*Urology*,” published in 1954 [6], which was not accompanied by a reference (Fig. 1).

Ever since, it has become dogma, taught to medical students and other medical professionals without being verified scientifically.

## Renewed concept

Thanks to considerable advancements in medical imaging technologies such as computed tomography (CT) and magnetic resonance imaging, detailed, three-dimensional information can now be obtained from a living individual without direct observation of organs and tissues. This dramatic development in radiological imaging has greatly contributed, of course, to quality improvements in clinical practice; still further, it enables the discovery of new anatomical structures that would have been difficult to recognize using conventional anatomical approaches.

**Fig. 2:**
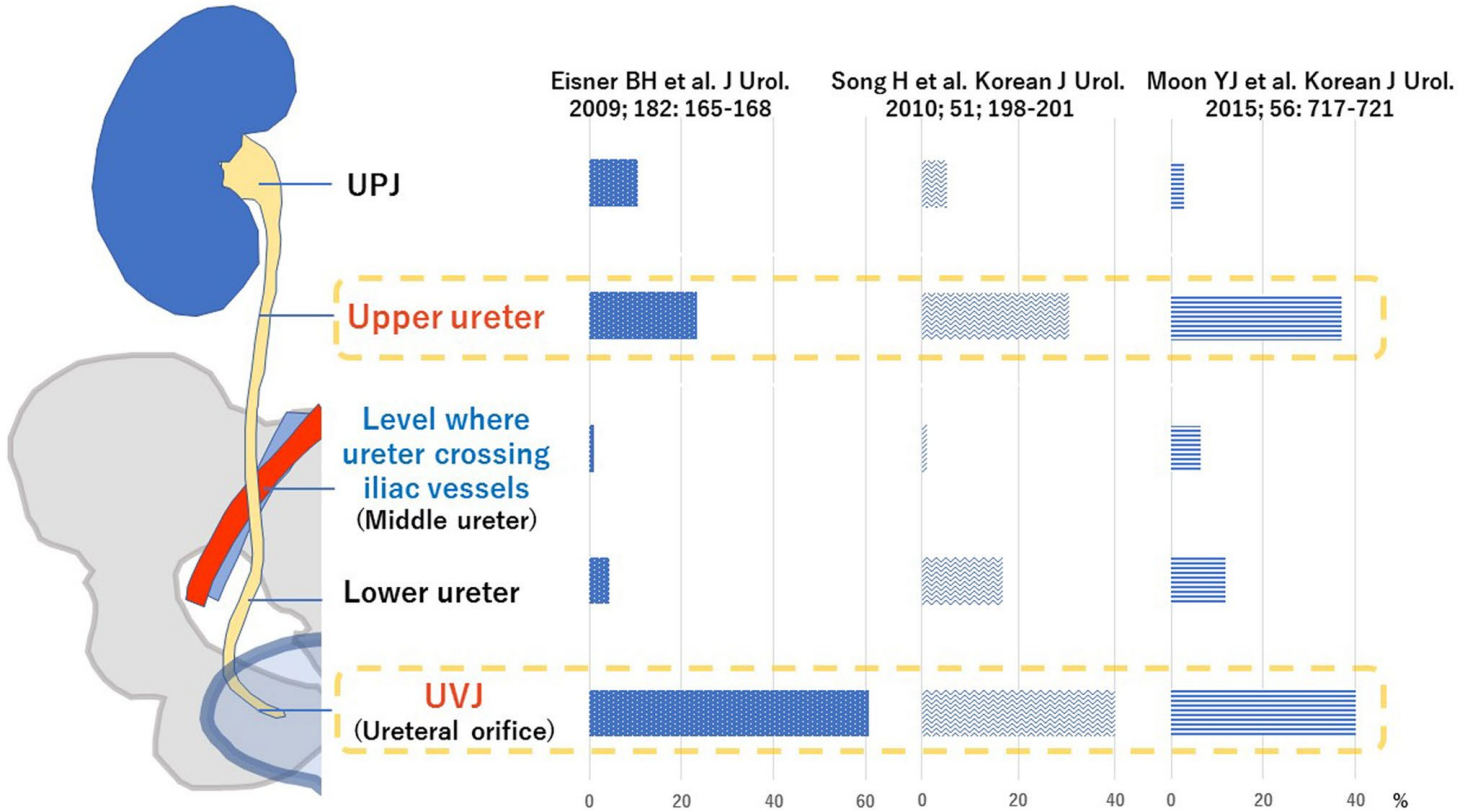
Distributions of upper urinary stone location assessed by computed tomography. Recent several studies have revealed that stones do not commonly lodge at the level where the ureter crosses the iliac vessels. The two peak sites where stones lodge in the upper urinary tract are the upper ureter and the ureterovesical junction (UVJ). *UPJ* Ureteropelvic junction. (The data presented in the figure are from Eisner et al. [7], Song et al. [8], and Moon et al. [9])

In 2009, Eisner et al. [7] located ureteral stones in patients with renal colic at an emergency department, using CT images. They revealed that there were only two peak sites of stone locations in the upper urinary tract: the upper ureter and the ureterovesical junction (UVJ). The level where the ureter crosses the iliac vessels was found not to be one of the common stone locations. Similar results were observed in several subsequent studies (Fig. 2) [8–10], bringing an end to the long-time conventional concept of three physiological narrowings in the upper urinary tract. Thus, the renewed concept defines only two physiological narrowings: those in the upper ureter and the UVJ.

## Structures of the retroperitoneum surrounding the ureter

The mechanism by which stones lodge at the level of the UVJ is well understood: the UVJ has a valve-like function to prevent urinary reflux, and the intravesical portion of the ureter is the narrowest section of the upper urinary tract [11]. Less well understood, on the other hand, are the factors that cause stones to lodge at the level of the upper ureter. We investigated the retroperitoneal anatomy in this area by correlating cadaveric findings with those of CT images; thereby, we revealed its mechanism and made a new anatomical discovery [12].

**Fig. 3:**
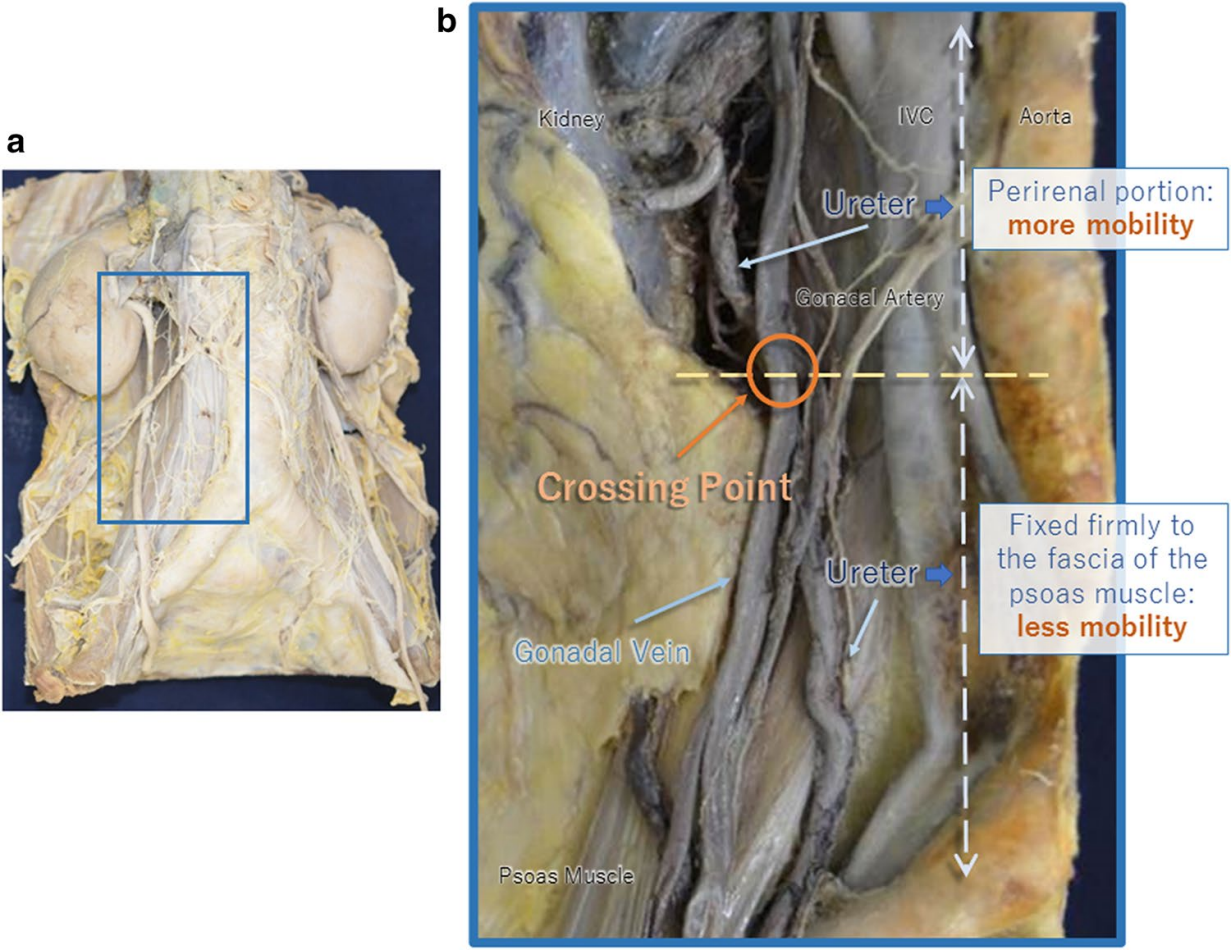
Photographs of the retroperitoneal region of a cadaveric specimen. **a** Macroscopic view. The retroperitoneum of the cadaver is fixed in formalin, and the intraperitoneal organs are removed. **b** Magnified view around the upper ureter. The ureter can be observed beneath the layer of gonadal vessels. The ureter is first located in the perirenal portion; thereafter, it is fixed firmly to the anteromedial aspect of the psoas major muscle. The level where the ureter exits the perirenal space approximately corresponds to the level where the ureter crosses under the ipsilateral gonadal vein. This point is termed the “crossing point.” (Cited and revised from [12])

The retroperitoneal region of a cadaveric specimen is displayed in Fig. 3. When approached from the ventral side, the ureter is observed beneath the layer of gonadal vessels. Continuing from the renal pelvis, the ureter is first located in the perirenal portion. This part of the ureter is relatively mobile within the perirenal fat pads. Where the ureter exits the perirenal space, it is fixed firmly to the anteromedial aspect of the psoas major muscle. This part of the ureter is less mobile. In other words, the ureter’s state of mobility and fixation changes at the point where it passes through the border of the perirenal space. We discovered that this occurs approximately at the level where the ureter crosses dorsal to the ipsilateral gonadal vein, and termed it the “crossing point” [12].

## Do urinary tract stones lodge at the crossing point?

**Fig. 4:**
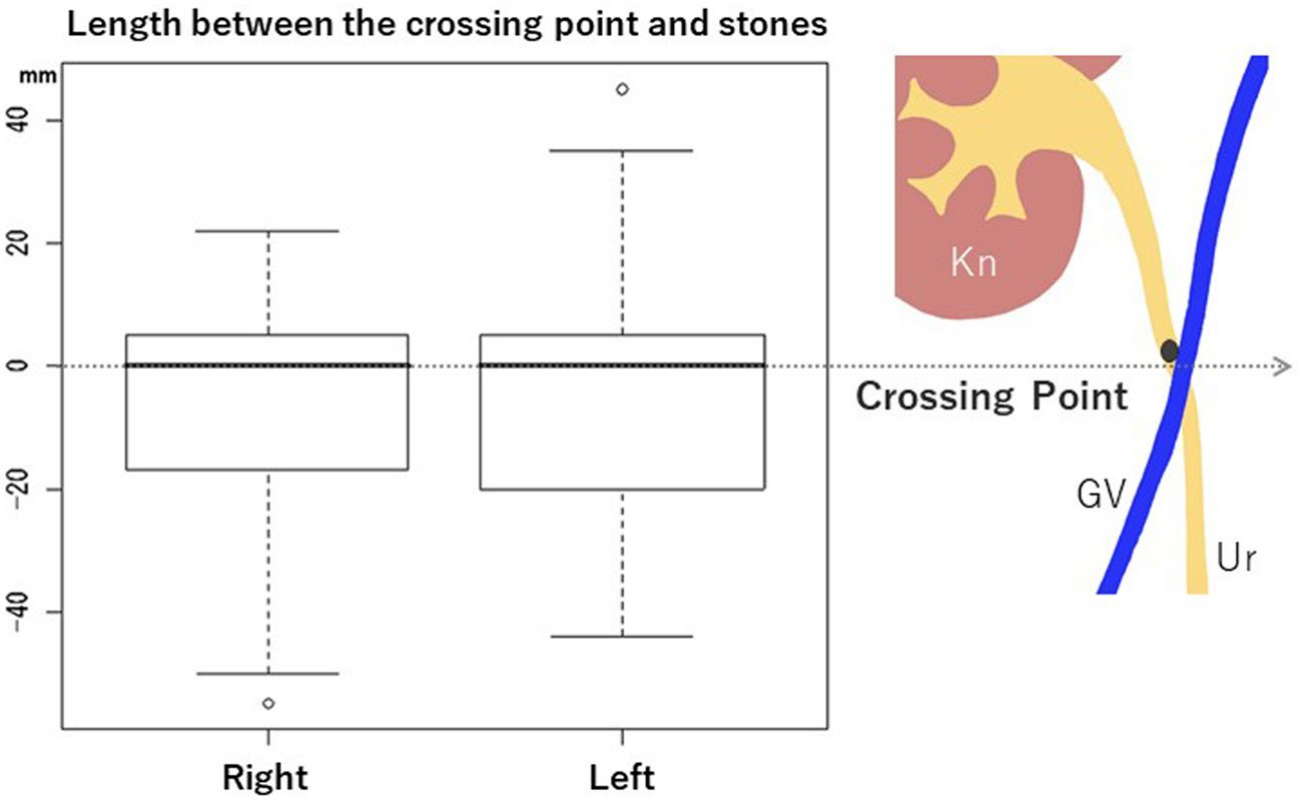
Box plot of the distance between the crossing point and stone location (as discovered in [13]). The peak site of urinary stone distribution in the upper ureter is corresponding approximately to the level of the crossing point on both sides. *Kn* Kidney, *GV* Gonadal vein, *Ur* Ureter

**Fig. 5:**
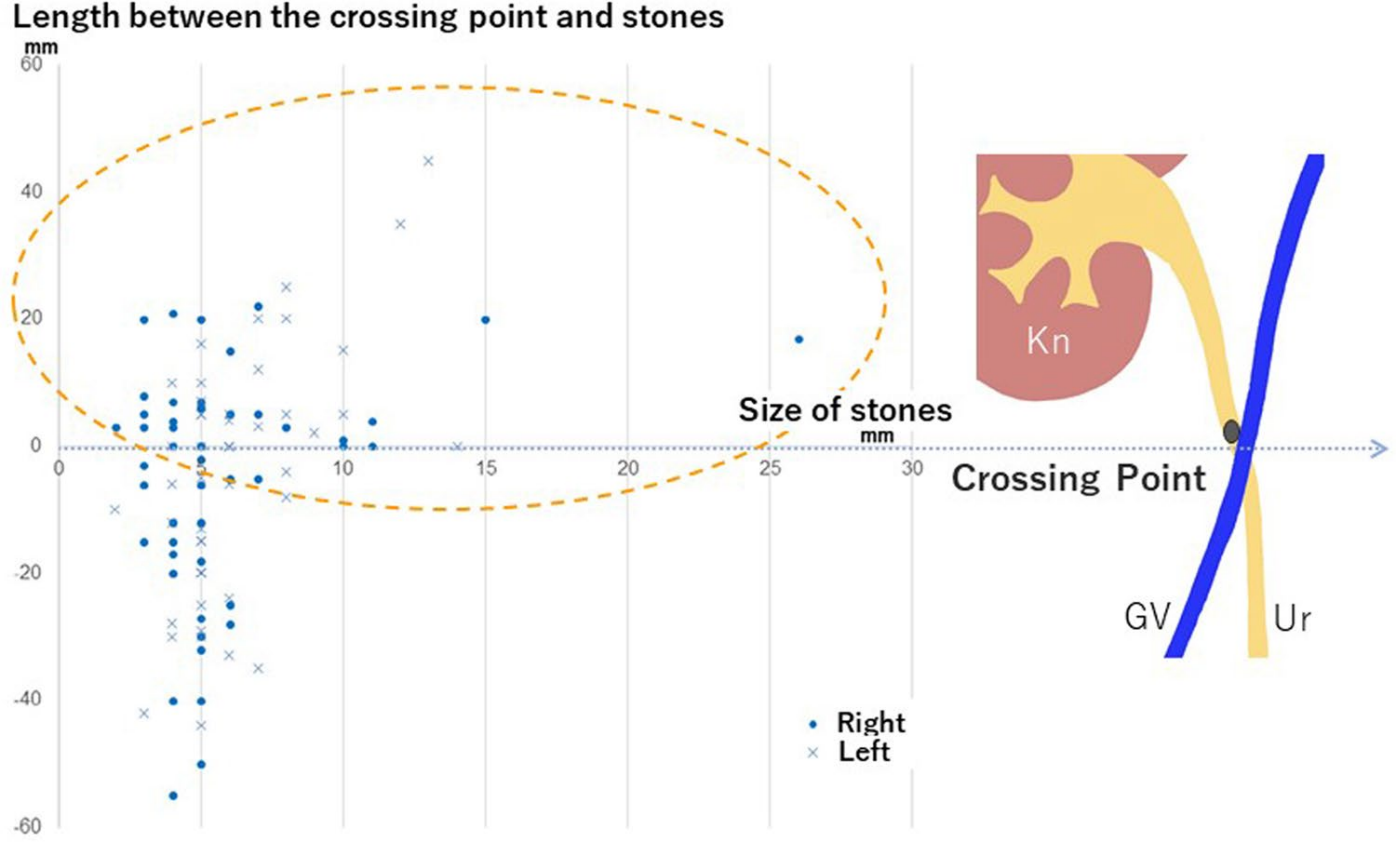
Distribution of stones, by size, relative to the crossing point (cited from [13]). No large stones are observed below the level of the crossing point. *Kn* Kidney, *GV* Gonadal vein, *Ur* Ureter

In another study, we investigated CT images of patients who presented to the emergency department with acute back pain and were diagnosed with acute renal colic [13]. We discovered that the peak site of urinary stone distribution in the upper ureter was corresponding approximately to the level of the crossing point (Fig. 4), which is due to the change in ureteral fixation at that level. It was also revealed that large stones tended to be located only at or above the level of the crossing point (Fig. 5). This endorses the crossing point as the location where not only ureteral mobility, but also ureteral compliance changes.

## Is the crossing point different from the ureteropelvic junction?

The upper urinary tracts are continuous tubes conveying urine from the kidneys to the bladder. They are covered with the same urothelium throughout, with no definite histological difference between the renal pelvis and the ureter. Thus, the definition of the ureteropelvic junction (UPJ) is based not on a histological, but on a morphological feature: the location where the diameter of the upper urinary tract shrinks. This is a somewhat subjective definition and, therefore, can be confusing.

**Fig. 6:**
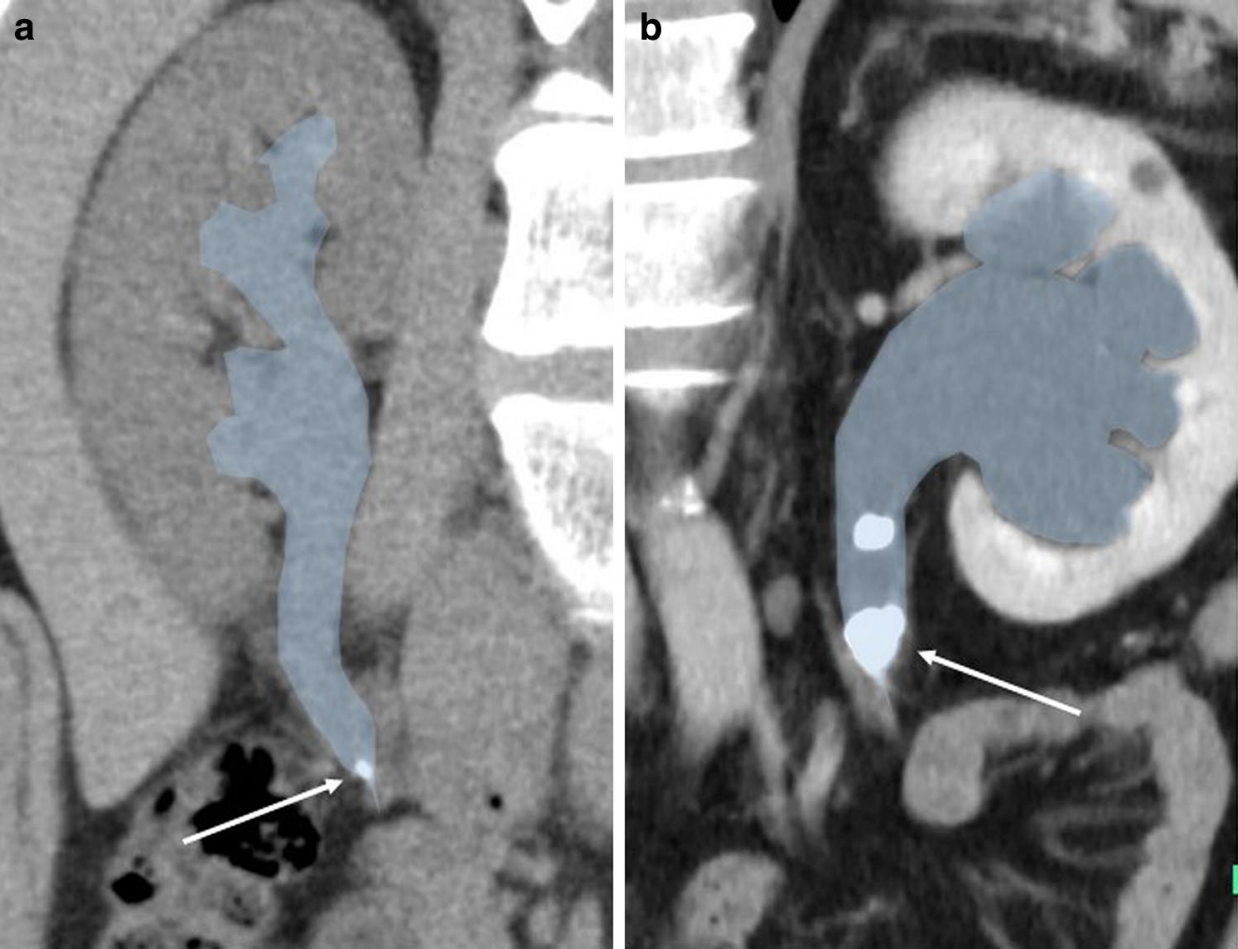
Oblique coronal computed tomography images of stones lodging at the level of the crossing point. **a** A 27-year-old male with a urinary stone at the level of the crossing point, **b** A 53-year-old male with a urinary stone at the level of the crossing point. When a stone lodges in the urinary tract (white arrows), the upstream tract dilates because of urinary stasis. Thereby, the crossing point mimics the ureteropelvic junction, the location where the diameter of the urinary tract originally changes

When a stone lodges in the urinary tract, the upstream tract dilates because of urinary stasis. If dilatation occurs at the level of the upper ureter or crossing point, its dilated diameter mimics that of the UPJ, even if it is not at the level where the diameter originally changes (Fig. 6). We suspect that this often leads to misidentification of the stone lodge site as the UPJ in clinical settings.

## Kinking of the upper ureter

**Fig. 7:**
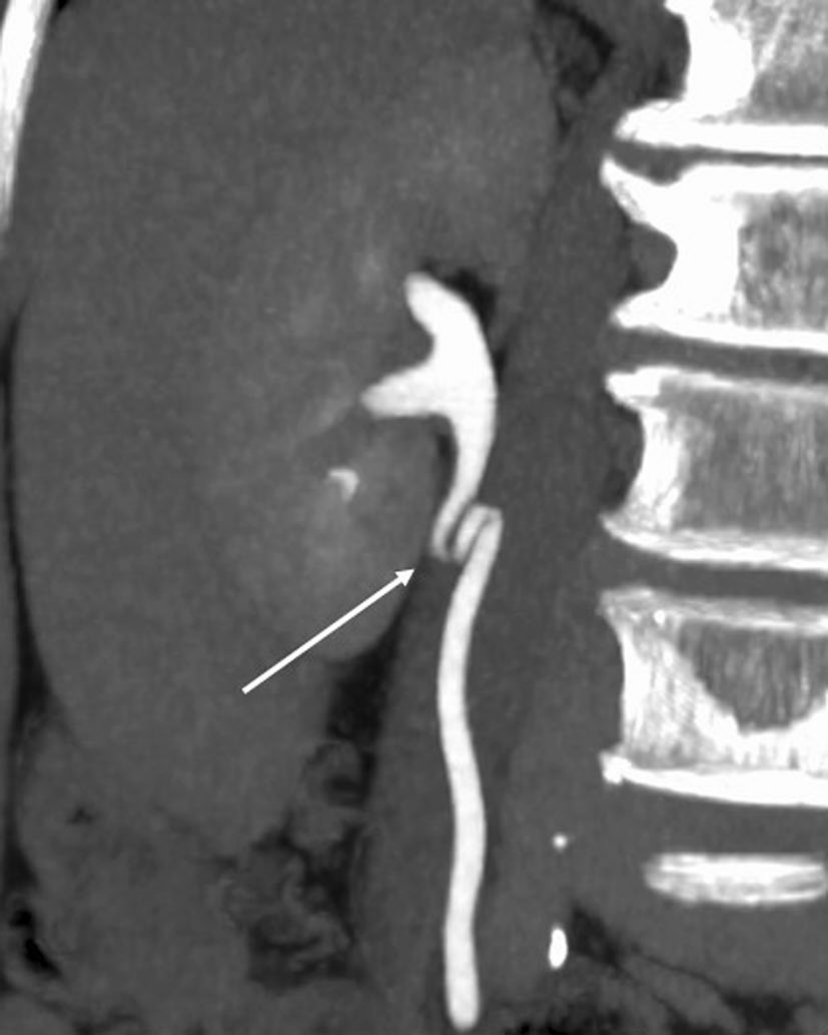
Kinking of the upper ureter in a reconstructed excretory coronal CT image of a 63-year-old male (cited from [12]). In retrograde pyelography or computed tomography (CT) urography, kinking of the upper ureter is not rare, although usually ignored

Kinking of the upper ureter is not a rare phenomenon and can be observed during retrograde pyelography or CT urography in daily clinical practice (Fig. 7). This finding is often ignored, probably because it is understood empirically by most radiologists or urologists that the kinking itself does not usually cause clinical problems. But in fact, this finding is another manifestation of the new anatomical concept of the retroperitoneal space aforementioned.

**Fig. 8:**
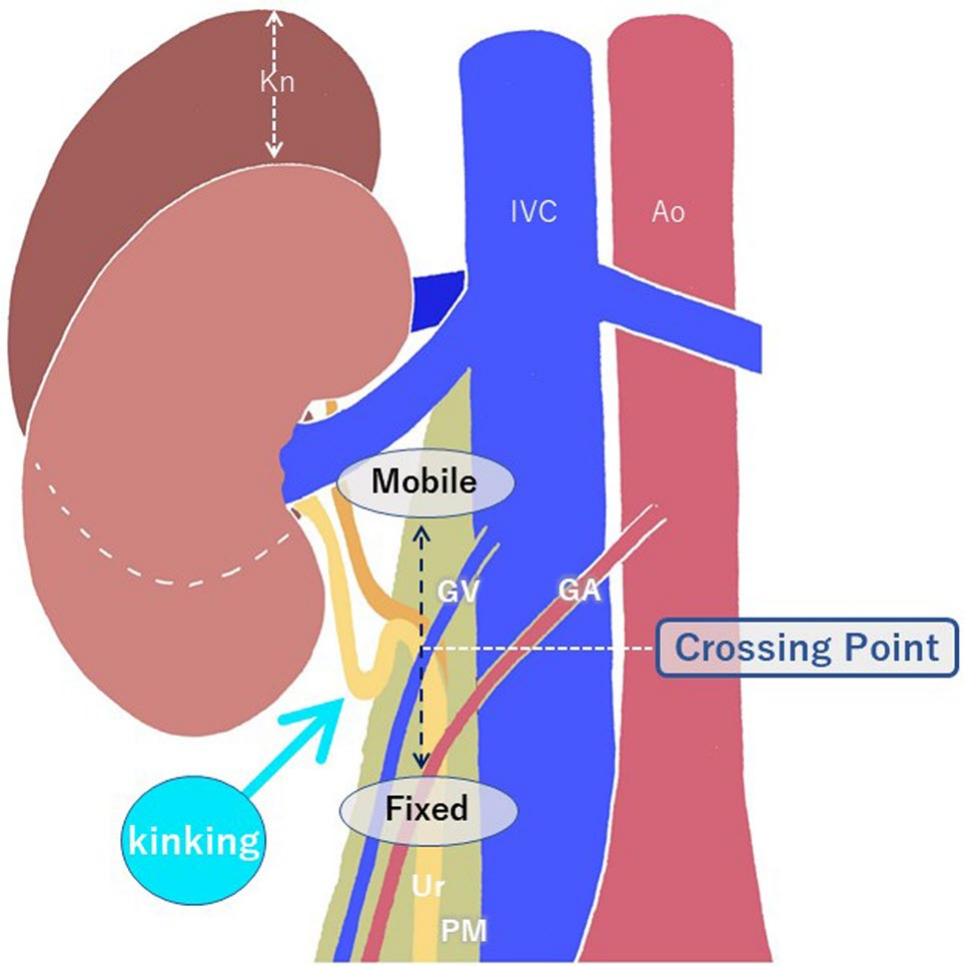
Underlying mechanism of kinking of the upper ureter (cited from [12]). The ureter is relatively mobile in the perirenal space (above the level of the crossing point), allowing it to loosen when the kidney descends. This phenomenon manifests as kinking of the ureter. *Kn* Kidney, *IVC* Inferior vena cava, *Ao* Aorta, *GV* Gonadal vein, *GA* Gonadal artery, *Ur* Ureter, *PM* Psoas muscle

**Fig. 9:**
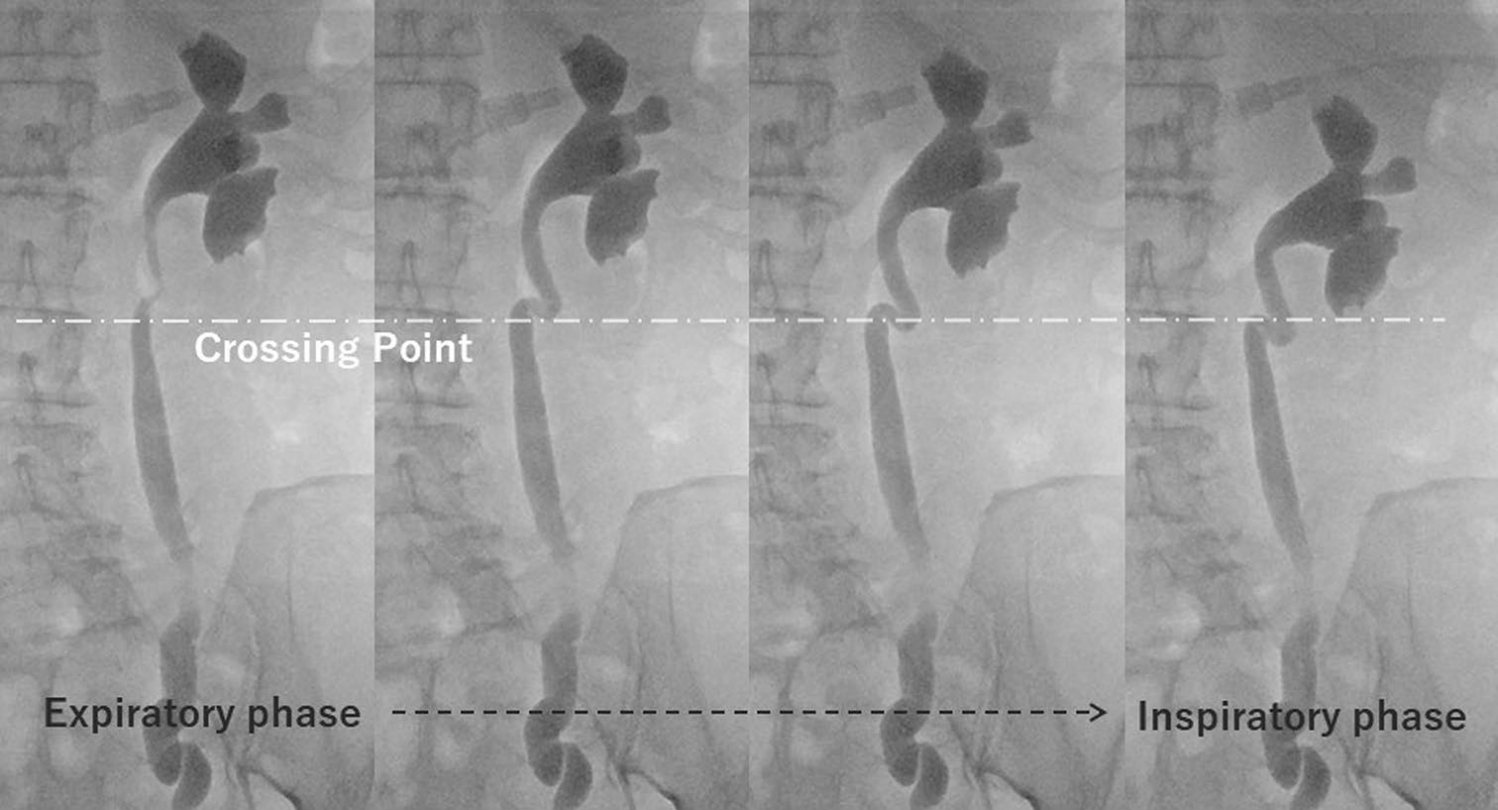
Fluoroscopic images of the dynamic movement of the upper ureter corresponding to that of the kidney. Angiography of a 73-year-old female with vaginal bleeding due to metastasis of ovarian cancer. Dynamic movement of the ureter correlating with that of the kidney is well visualized in fluoroscopy. Kinking arises when the kidney descends during the inspiratory phase, resulting from loosening of the upper ureter

Kinking of the upper ureter is identified in approximately 20% of CT urography images [12]. It occurs always at or above the level of the crossing point. The underlying mechanism is the relative mobility of the ureter in the perirenal space (above the level of the crossing point), allowing it to loosen when the kidney descends by respiration or other physiological movements. This physiological phenomenon is radiologically identified as kinking of the ureter (Fig. 8). In other words, this finding reflects the fact that the ureter is always forced to adjust its length according to the kidney’s dynamic movement in the retroperitoneum. Additionally, it should be mentioned that CT urography is usually performed during the inspiratory phase, which is when the kidney descends; this theoretically increases the likelihood of observing kinking of the ureter. Dynamic movement of the ureter correlating with that of the kidney can also be observed during angiography or fluoroscopy (Fig. 9), which enhances understanding of the structural dynamics of this phenomenon. During hydronephrosis caused by obstruction in the lower ureter, this kinking is even more prominent.

In short, although kinking of the upper ureter itself is a normal radiological finding, it is an important key to understand the whole picture of the underlying retroperitoneal anatomy around the ureter.

## Clinical relevance and perspective

This novel retroperitoneal anatomical concept has brought about a paradigm shift in the conventional theory of the physiological narrowings in the upper urinary tract. In CT diagnosis of urinary stones obstruction, it is essential to recognize this new concept and assess stone location correctly. In future, the clinical characteristics and prognosis of stones lodging at the crossing point should be determined. This anatomical discovery also has the potential to bring about new surgical approaches and better understanding of the retroperitoneal space. In addition, further investigation is needed to assess the relationship between the site of conventionally known as “UPJ obstruction” and the crossing point.

## Conclusion

1. There are only two physiological narrowings in the upper urinary tract: the upper ureter and the UVJ.
2. Urinary stones commonly lodge in the upper ureter due to the change in ureteral mobility and compliance where the ureter exits the perirenal space.
3. The level of this common obstruction site is at the crossing point, where the ureter crosses over the ipsilateral gonadal vein.
4. Kinking of the upper ureter is a normal finding, caused by loosening of the upper ureter at or above the crossing point.
